# Estimation of healthcare‐related charges in women with *BRCA* mutations and breast cancer

**DOI:** 10.1186/s12913-020-06038-z

**Published:** 2021-01-13

**Authors:** Joseph Biskupiak, Sudhir Unni, Claire Telford, Minkyoung Yoo, Xiangyang Ye, Rishi Deka, Diana Brixner, David Stenehjem

**Affiliations:** 1 Department of Pharmacotherapy, Outcomes Research Center, University of Utah, Salt Lake City, USA; 2 Daiichi-Sanyko Inc, Baskin Ridge, New Jersey, Utah USA; 3 GSK, Gaithersburg, Maryland USA; 4 University of California San Diego, La Jolla, California USA; 5 University of Minnesota, Minneapolis, Minnesota USA

**Keywords:** Breast cancer, BRCA, HER-2, Charges

## Abstract

**Background:**

Breast cancer costs were estimated at $16.5 billion in 2010 and were higher than other cancer costs. There are limited studies on breast cancer charges and costs by BRCA mutations and receptor status. We examined overall health care and breast cancer-related charges by BRCA status (BRCAm vs. BRCAwt), receptor status (HER2+ vs. HER2-), and treatment setting (neoadjuvant vs. adjuvant).

**Methods:**

Retrospective cohort study of charge data from 1995-2014 in an academic medical center. Facilities, physician, pharmacy, and diagnosis-related charges were presented as mean and median charges with standard deviation (SD) and interquartile ranges (25%-75%). Wilcoxon rank-sum test was used to assess statistically significant differences in charges between comparators.

**Results:**

Total median breast-cancer related charges were $65,414 for BRCAm and $54,635 for BRCAwt (*p*=0.19); however all-cause charges were higher for BRCAm patients ($145,066 vs. $119,119, *p*<0.001). HER2+ status was associated with higher median breast cancer charges ($152,159 vs. $44,087, *p*<0.0001) that was driven by the charges for biological agents. Patients initially seen in the neoadjuvant setting had higher mean breast cancer charges than in the adjuvant setting ($117,922 vs. $80,061, *p*<0.0001).

**Conclusion:**

BRCA mutation status was not associated with higher breast cancer charges but HER2+ status had significantly higher charges, due to charges for biological agents. Patients who initially received neoadjuvant treatment had significantly higher overall treatment charges than adjuvant therapy patients. With the advent of novel therapies for *BRCA*m, the economic impact of these treatments will be important to consider relative to their survival benefits.

## Background

Breast cancer is the most common type of cancer observed in women in the US and is associated with significant clinical and economic burden [1]. In 2015, there were nearly 3.5 million women living in the US with a breast cancer diagnosis and it is estimated that approximately 12.4% of women in the US will receive a breast cancer diagnosis at some point in their lives [1].

Breast cancer is associated with significant costs. In 2010, breast cancer treatment costs in the US were approximately $16.5 billion, which was higher than those for any other cancer and these costs are expected to rise to $20.5 billion by 2050 [2]. Similar results have also been observed in Europe wherein breast cancer costs accounted for the highest healthcare costs of all cancers [3]. The published economic studies in breast cancer vary widely by perspective, methodology, time horizon, and patient populations [4]. While breast cancer costs have been estimated by tumor stage, time after diagnosis, and treatment options, there are limited studies on such costs categorized by BRCA mutations and receptor status as well as neoadjuvant and adjuvant treatment settings [4–6].

The assessment of costs within a healthcare system is challenging given the complex interplay between costs, charges, and reimbursed amounts for the service provided [7]. In many instances, it is difficult to access costs due to their proprietary nature, which is dependent on the contractual agreements between the healthcare systems and payer organizations. Studies have used cost-to-charge ratios, the ratio of charges over CMS allowable costs, to describe the costs assigned by the healthcare system. However, there are significant variations in these cost estimates depending on the type of institution and geographical location [8, 9]. Thus, healthcare studies typically use the more readily available charge data to make economic assessments.

The purpose of this study was to explore cumulative health care charges in women diagnosed with breast cancer and tested for BRCA mutations with charges stratified by BRCA mutation status, receptor status, and treatment settings.

## Methods

### Study design, population, and data source

This was a retrospective cohort study utilizing charge data from 1995 to 2014 in the University of Utah Clinical Enterprise Data Warehouse (EDW), a repository containing records for more than 3.3 million patients dating back to 1995. The EDW contains data from electronic medical records, laboratory and radiology findings, administrative claims, and patient encounter data. Patients included in the study were adult women with breast cancer who were tested for BRCA mutations (BRCA positive [BRCAm] or BRCA wildtype [BRCAwt]) and received treatment at the Huntsman Cancer Institute (HCI) in Salt Lake City, Utah. HCI is the major cancer center in the Intermountain West region as well as a National Cancer Institute-Designated Cancer Center and member of the National Comprehensive Cancer Network. Patients were identified from the Huntsman Cancer Institute Tumor Registry (HCI-TR) using site (ICD-O C50.x) and histology codes for the diagnosis of breast cancer between January 1, 1995 and December 31, 2014. To our knowledge, healthcare providers from HCI had unrestricted availability to request BRCA testing during the study period. The date of breast cancer diagnosis was defined as the index date. Electronic medical records of eligible patients were manually reviewed to identify and obtain information regarding BRCA mutation, HER-2, and ER/PR status, treatment patterns, and disease progression. Chart abstraction was conducted by an oncology clinical pharmacist using a chart abstraction form and reviewed by another oncology clinical pharmacist. Demographic and clinical characteristics of these patients were obtained through a EDW query.

Patients were excluded from the study if they were < 18 years of age at the time of breast cancer diagnosis, had < 2 encounters (clinic visits) separated by ≥ 30 days with relevant ICD-9 codes for breast cancer in the EDW, were male patients, were not tested for a BRCA mutation or had unknown BRCA mutation status, or had no charge data.

The charge amount was the actual amount charged by the facility based on the International Classification of Diseases, 9th Clinical Modification (ICD-9-CM) diagnosis and Current Procedural Terminology (CPT) codes. The estimated yearly charges were adjusted for inflation to 2014 values using the Bureau of Labor Statistics Consumer Price Index for Medical Care [10].

### Study outcomes

The patients identified in this study were linked to the administrative data in the EDW to obtain all charge data related to the healthcare utilization from the index date until they were lost to follow-up (no additional encounters in the database or death occurred). Charges (the monetary amount billed for services provided to a patient) were initially categorized based on paid claims linked to the corresponding ICD-9 and CPT codes as breast cancer-related, other (non-breast) cancers related, non-cancer related, and all-cause charges. Each categorical charge was stratified as inpatient- and outpatient-related charges based on site of care. Inpatient charges refer to charges incurred whn a patient has been admitted to a hospital for care. Outpatient charges refer to charges incurred when a patient is treated in an ambulatory clinic setting (not admitted to a hospital for care). All-cause charges were defined as the sum of all charges (breast cancer-related, other cancers related, and non-cancer related) incurred from index date to death or the end of study whichever occurred earlier.

Charges were also categorized as facilities/technical charges, physician/professional charges, and pharmacy charges. The pharmacy charges were sub-divided by the type of treatment provided (chemotherapy, hormonal therapy, biological therapy, targeted small molecule therapy, investigational drug, chemotherapy administration charges, and other medications).

Breast cancer-related charges were further stratified by BRCA mutation status, HER2 status, and initial treatment setting at cancer diagnosis. Initial treatment setting was defined as the first treatment for the breast cancer patient at diagnosis and included non-metastatic adjuvant therapy, non-metastatic neoadjuvant therapy, surgical resection, and treatment for metastatic disease. BRCA mutation status was categorized as BCRA mutated gene (BRCAm) and BCRA wild-type gene BCRAwt. HER2 status was categorized as HER2+ (HER-2 amplified tumors) that included ER+/PR+/HER2 + and ER-/PR-/HER2 + patients; and HER2- (HER2 non-amplified tumors) that included ER-/PR-/HER2- (also called triple negative breast cancer [TNBC]) and ER+/PR+/HER2- patients. If a patient progressed to metastatic disease after receiving adjuvant, neoadjuvant or surgical resection, their charges were assigned to the initial treatment setting until progression to metastatic disease at which point subsequent charges were assigned to the metastatic group. Hence, metastatic patients could contribute charges to more than one treatment setting; however, their charges were not double counted.

### Statistical analysis

Charges were presented as mean and median charges with standard deviation (SD) and interquartile ranges (25%-75%). Wilcoxon rank-sum test was used to assess statistically significant difference in health care charges across comparator groups. All analyses were conducted using SAS version 9.3 (SAS Institute, Cary, NC).

## Results

**Fig. 1:**
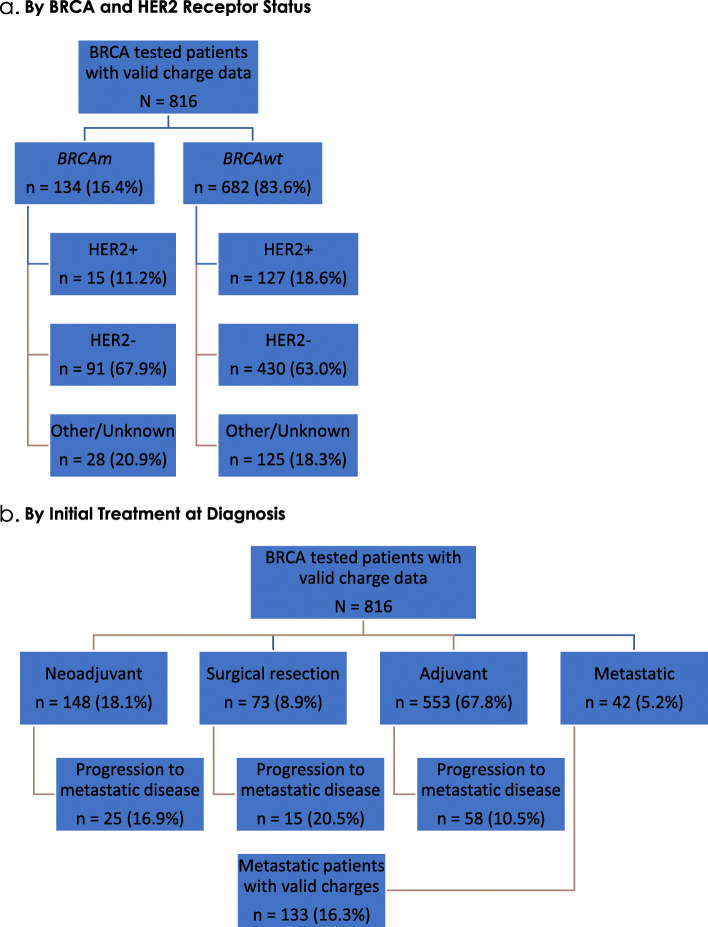
Patient Identification Flow Chart . **a**. By BRCA and HER2 Receptor Status. **b**. By Initial Treatment at Diagnosis

A total of 5,712 women had a diagnosis of breast cancer during 1995–2014 of which 835 (14.6%) were tested for a BRCA mutation with documented results from BRCA testing (Myriad Laboratories, Inc.) The number of women undergoing BRCA testing increased from 2000 to 2014. When categorized by 5-year increments by year of diagnosis, 5.7% women were tested during 2000–2004, 17.1% were tested during 2005–2009, and 23.1% were tested during 2010–2014. Of the 835 patients with BRCA testing, 816 had valid charge data and were included in the study. There were 134 (16.4%) patients with a BRCAm mutation and 682 (83.6%) patients were BRCAwt. Within these groups, HER2 + tumors were observed in 15 (11.2%) and 127 (18.6%) patients; HER2- tumors were seen in 91 (67.9%) and 430 (63.0%) patients; and 28 (20.9%) BRCAm and 125 (18.3%) BRCAwt patients had other/unknown status (Fig. 1a).

With respect to initial treatment setting after breast cancer diagnosis, 553 (67.8%) patients received adjuvant therapy, 148 (18.1%) patients received neoadjuvant therapy, and 73 (8.9%) patients had surgical resection alone. There were 42 (5.1%) patients who presented with metastatic breast cancer at the time of diagnosis. Of the patients who received adjuvant, neoadjuvant and surgical resection at diagnosis, 58 (10.5%), 25 (16.9%), and 15 (20.5%) progressed to metastatic disease during the study period respectively. The median time to progression for these 98 patients was 3.5 years (± IQR 2.2–9.7 years). Thus, of the total 140 patients who had metastatic disease during the study period, 133 (16.3% of 816 patients) had valid charges (Fig. 1b).

Table 1 lists the demographic and clinical characteristics of these patients. Age at diagnosis was similar between BRCAm and BRCAwt (45.9 years vs. 47.1 years) patients; breast cancer stage at diagnosis was also similar between the two groups. BRCAm patients had significantly longer median follow-up times (62.4 [± 74.5] months vs. 46.6 [± 57.7] months, *p* = 0.0045), The BRCAm group had more triple negative breast cancer patients vs. BRCAwt group (28.4% vs. 11.9%). More BRCAm patients had grade 3 histological tumor grades and conversely, more BRCAwt patients had grade 2 histology (Grade 1: 52.2% vs. 32.0%; Grade 2: 38.1% vs. 45.0%).

**Table 1:** Demographic and Clinical Characteristics of the Study Sample

Variables	***BRCA***m (***n***=134)	***BRCA***wt (***n***=682)	***BRCA***m vs. ***BRCA***wt ***p***-value
**Demographic Characteristics**			
**Age,** Mean ± SD	45.9 ± 9.4	47.1 ± 11.7	0.2575^a^
**Age,** Median ± IQR	45.0 ±12.9	45.9 ± 14.7	0.3853^b^
**Ethnicity**			
Caucasian/ White	116 (86.6%)	598 (87.5%)	0.9377^c^
Non-White	10 (7.5%)	47 (6.9%)	
Unknown	8 (6.0%)	37 (5.4%)	
**Plan Type**			
Commercial	93 (69.4%)	426 (62.5%)	0.1324^c^
Medicare	6 (4.5%)	60 (8.8%)	
Medicaid	9 (6.7%)	31 (4.6%)	
Other/Unknown	26 (19.4%)	165 (24.2%)	
**Clinical Characteristics**			
**Stage at diagnosis**			
I	51 (38.1%)	262 (38.4%)	0.7387^a^
II	51 (38.1%)	250 (36.7%)	
III	16 (11.9%)	107 (15.7%)	
IV	9 (6.7%)	34 (5.0%)	
Unknown	7 (5.2%)	29 (4.3%)	
**Reeptor status**			
ER+/PR+/HER2-	53 (39.6%)	349 (51.2%)	**<0.0001**^a^
ER+/PR+/HER 2+	7 (5.2%)	87 (12.8%)	
TNBC	38 (28.4%)	81 (11.9%)	
ER-/PR-/HER2+	8 (6.0%)	40 (5.9%)	
Other/Unknown	28 (20.9%)	125 (18.3%)	
**Tumor histologic grade**			
1	7 (5.2%)	115 (16.9%)	**<0.0001**^a^
2	51 (38.1%)	307 (45.0%)	
3	70 (52.2%)	218 (32.0%)	
Unknown	6 (4.5%)	42 (6.2%)	

*SD* Standard Deviation, *IQR* Interquartile Range, *TNBC* Triple Negative Breast Cancer (ER-/PR-/HER2-)

^a^T-Test

^b^Wilcoxon Rank Sum Test

^c^Chi-Squared Test

### Comparison of health care cumulative charges between BRCAm and BRCAwt patients

**Fig. 2:**
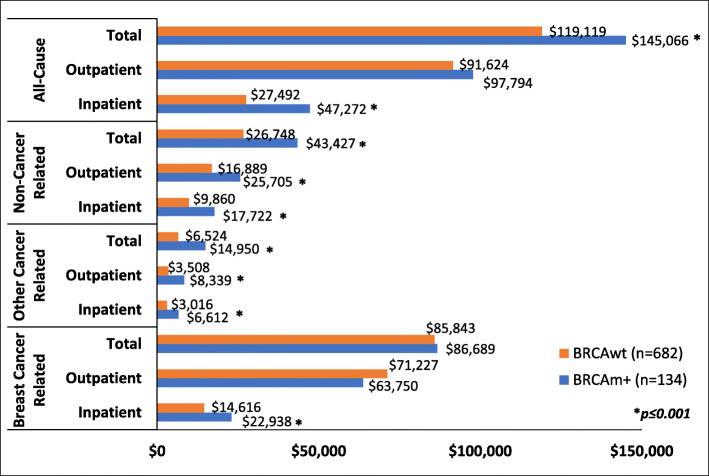
Mean Cumulative Health Care Charges by BRCA Status

Total mean (SD) breast cancer related charges were similar between BRCAm vs. BRCAwt patients, $86,689 ($75,937) vs. $85,843 $97,304, *p* = 0.19, respectively (Table 2) while all-cause charges were significantly higher for BRCAm patients ($145,066 [$117,462] vs. $119,119 [$122,169], *p* < 0.001; Fig. 2). Facility/technical charges accounted for 40.5% and 32.5% of the total breast cancer related charges for BRCAm and BRCAwt groups (Table 2). Pharmacy charges accounted for 40.8% and 49.4% of total breast cancer charges for BRCAm and BRCAwt groups (Table 2). Chemotherapy was received by 58% of patients and accounted for 23.9% of pharmacy charges for BRCAm patients; biologics were received by 16% of patients and accounted for 43.0% of pharmacy charges for BRCAwt patients. Physician/professional charges accounted for 18.7% and 18.1% of the total breast cancer related charges for BRCAm and BRCAwt groups respectively (Table 2).

**Table 2:** Breast Cancer Related Cumulative Charges by BRCA Status and Charge Type

Health care charges ($US)	Overall ***n***=816		BRCAm ***n***=134		BRCAwt ***n***=682		BRCAm vs. BRCAwt
	Mean (SD)	Median (IQR)	Mean (SD)	Median (IQR)	Mean (SD)	Median (IQR)	***p***-value
**Facilities/ Technical**	29,090 (26,802)33.8%^a^	24,027 (34,043)	35,130 (28,878) 40.5%	33,037 (42,145)	27,903 (26,234) 32.5%	22,458 (32,529)	**0.01**
**Physician/ Professional**	15,615 (13,126), 18.2%	12,441 (17,669)	16,207 (12,198), 18.7%	15,624 (18,967)	15,498 (13,306) 18.1%	11,908 (16,906)	0.25
**Pharmacy**	41,277 (69,308) 48.0%	9,313 (58,716)	35,352 (51,919) 40.8%	11,379 (54,399)	42,442 (72,209) 49.4%	8,930 (59,659)	0.65
**Total Anticancer** **Treatment**	24,616 (56,779)	2,018 (15,804)	16,821 (38,088)	2,756 (14,673)	26,148 (59,671)	1,824 (15,995)	0.72
Other Medication(s)	16,661 (24,471)	2,911 (27,834)	18,531 (24,484)	6,035 (30,729)	16,294 (24,470)	2,609 (26,434)	0.19
Clinical Trial	56 (347)	0 (0)	89 (621)	0 (0)	49 (262)	0 (0)	0.93
Chemotherapy	7,171 (14,088)	1,372 (6,575)	8,439 (14,791)	2,344 (9,463)	6,922 (13,944)	1,180 (6,124)	0.13
Hormonal Therapy	890 (5,521)	0 (0)	769 (4,334)	0 (0)	914 (5,728)	0 (0)	0.53
Biologic Medicine	16,499 (50,091)	0 (0)	7,524 (30,898)	0 (0)	18,262 (52,891)	0 (0)	**0.02**
Chemotherapy Administration	0 (5)	0 (0)	1 (7)	0 (0)	0 (5)	0 (0)	NA
**Total Charges**	85,982 (94,087)	55,230 (94,787)	86,689 (75,937)	65,414 (95,894)	85,843 (97,304)	54,635 (94,085)	0.19

^a^Percent contribution of the charge type to total charges

*SD* Standard deviation, *IQR* Interquartile range

### Comparison of health care cumulative charges between HER2 + and HER2- patients

**Fig. 3:**
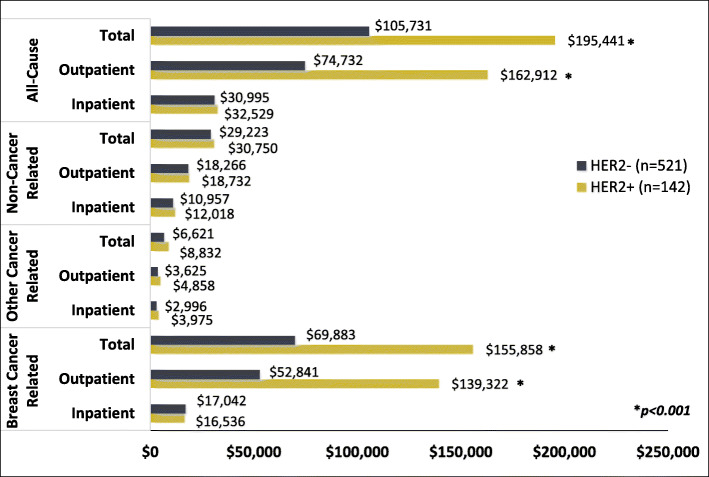
Mean Cumulative Health Care Charges by Receptor Status

Breast cancer related charges were significantly higher for HER2 + patients vs. HER2- ($155,858 vs. $69,883; *p* < 0.001, Fig. 3) which was primarily driven by outpatient charges ($139,322 vs. $52,841, *p* < 0.001, Fig. 3). In addition, HER2 + patients incurred significantly higher charges across nearly all breast cancer related charge categories (Table 3), especially biologic therapy ($71,855 [$72,075] vs. $732 [$12,692], *p* < 0.0001; Table 3).

**Table 3:** Breast Cancer Related Cumulative Charges by Receptor Status and Charge Type

Health care charges ($US)	HER2 + ER+/PR+/HER2 + or ER-/PR-/HER2 + ***n*** = 142		HER2- TNBC or ER+/PR+/HER2- ***n*** = 521		HER2 + vs. HER2-
	**Mean** **(SD)**	**Median** **(IQR)**	**Mean** **(SD)**	**Median** **(IQR)**	*p-value*
**Facilities/ Technical**	36,523 (28,082)	34,647 (39,683)	28,707 (26,418)	24,207 (32,460)	**0.00**
**Physician/ Professional**	18,191 (14,195)	16,138 (17,411)	15,648 (12,973)	12,577 (17,458)	**0.04**
**Pharmacy**	101,145 (94,243)	10,1374 (147,132)	25,529 (39,646)	7,303 (42,884)	**< 0.0001**
**Total Anticancer Treatment**	82,890 (81,269)	81,783 (127,300)	7,929 (21,782)	1,364 (6,006)	**< 0.0001**
Clinical Trial	96 (430)	0 (0)	39 (199)	0 (0)	0.26
Chemotherapy	10,443 (15,582)	4,417 (14,415)	6,222 (13,642)	1,074 (5,461)	**< 0.0001**
Hormonal Therapy	496 (3,889)	0 (0)	935 (5,660)	0 (0)	0.46
Biologic Therapy	71,855 (72,075)	6,5471 (117,006)	732 (12,692)	0 (0)	**< 0.0001**
**Other Medication(s)**	18,255 (21,988)	6,517 (29,473)	17,600 (25,321)	2,953 (29,228)	**0.02**

*SD *Standard deviation, *IQR *Interquartile range

### Comparison of health care cumulative charges by initial treatment setting

**Fig. 4:**
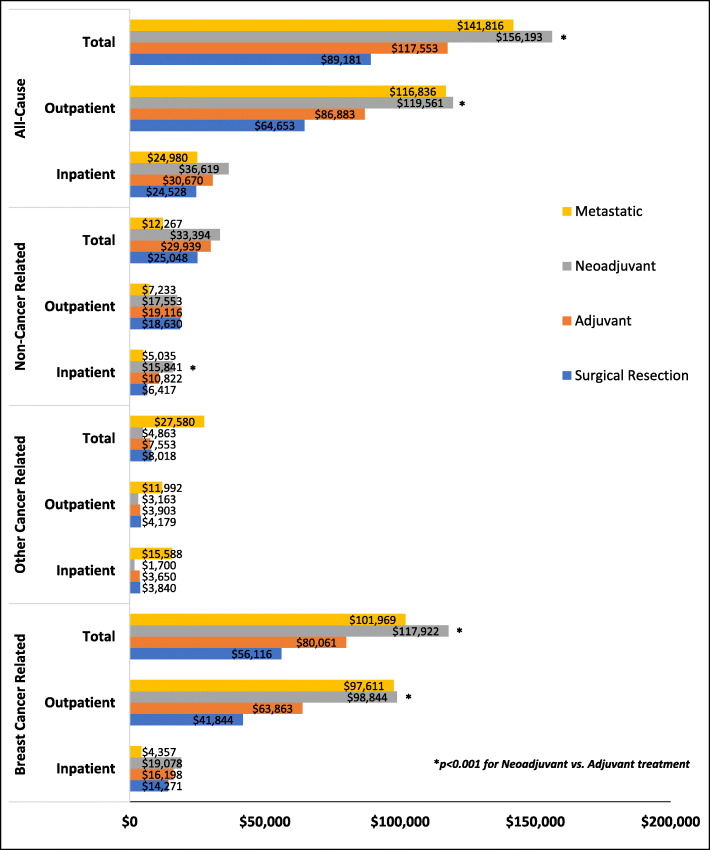
Mean Cumulative Health Care Charges by Initial Treatment Setting

Patients seen initially in the neoadjuvant setting had higher mean breast cancer related charges vs. those seen in the adjuvant treatment setting ($117,922 vs. $80,061, *p* < 0.0001; Fig. 4). Patients receiving neoadjuvant treatment had significantly higher charges across most of the charge categories vs. patients receiving adjuvant treatment (Table 4). Patients with metastatic disease had a mean breast cancer related total charge of $101,969 (Fig. 4).

**Table 4:** Breast Cancer Total Cumulative Charges by Initial Treatment at Diagnosis and Charge Type

Health care charges ($US)	Surgical Resection ***n***=73		Adjuvant ***n***=553		Neoadjuvant ***n***=148		Adjuvant vs. Neoadjuvant
	Mean (SD)	Median (IQR)	Mean (SD)	Median (IQR)	Mean (SD)	Median (IQR)	***p***-value
**Breast Cancer related**							
**Facilities/ Technical**	23,472 (21,512)	19,840 (28,345)	27,486 (24,400)	22,456 (32,113)	35,877 (27,496)	33,984 (38,937)	**0.00**
**Physician/ Professional**	12,942 (10,770)	10,840 (12,678)	15,575 (13,091)	12,449 (17,812)	17,340 (12,663)	15,536 (16,820)	**0.04**
**Pharmacy**	19,702 (57,840)	993 (2,650)	37,000 (66,360)	8,531 (51,815)	64,705 (79,214)	43,009 (83,327)	**<0.0001**
**Total Anticancer Treatment**	11,491 (43,165)	0 (0)	21,564 (53,960)	2,241 (13,526)	40,941 (71,342)	4,556 (57,935)	**0.00**
Clinical Trial	47 (196)	0 (0)	31 (215)	0 (0)	78 (297)	0 (0)	**0.01**
Chemotherapy	3,604 (12,107)	0 (0)	7,204 (14,053)	1,759 (6,352)	7,376 (10,451)	3,905 (9,278)	**0.01**
Hormonal Therapy	1,022 (5,843)	0 (0)	836 (5,814)	0 (0)	399 (2,711)	0 (0)	0.95
Biologic Medicine	6,818 (36,636)	0 (0)	13,492 (46,279)	0 (0)	33,088 (66,386)	0 (40,885)	**<0.0001**
Chemotherapy Administration	2 (15)	0 (0)	0 (4)	0 (0)	0 (0)	0 (0)	.
**Other Medication(s)**	8,210 (21,536)	993 (2,559)	15,436 (23,472)	2,530 (24,473)	23,764 (24,934)	12,018 (42,041)	**<0.0001**

*SD* Standard deviation, *IQR* Interquartile range

## Discussion

The purpose of this study was to identify the important drivers of charges in women with breast cancer who were tested for BRCA mutation status and received treatment at a National Comprehensive Cancer Network/National Cancer Institute (NCCN/NCI)-accredited comprehensive cancer treatment center. In addition to BRCA status, the study also categorized patients by the HER2 status and type of treatment received at the time of breast cancer diagnosis. The study outcomes included comparison of breast cancer related charges between BRCAm and BRCAwt patients; HER2 + and HER2- patients; patients receiving adjuvant and neoadjuvant treatments overall, and by charge type. The study also compared the breast cancer related charges vs. charges associated with other cancer types and non-cancer related charges in these patients.

Our study suggests that BRCAm patients had significantly higher all-cause charges vs. BRCAwt patients. While overall breast cancer related charges were similar between these patients, other cancer related charges were significantly different (mean [SD] $14,950 [$40,801] vs. $6,524 [$25,395], *p* < 0.0001). This difference in charges could potentially be attributed to risk-reducing surgical treatments or the development of other non-breast cancers including ovarian cancer and fallopian tube cancer. Also, BRCAm patients had nearly 16 months longer duration of follow-up vs. BRCAwt patients. We are unaware of any other studies assessing economic burden of any cancer based on BRCA mutation status. Thus, clinicians, health plans, and researchers may need to consider the difference in follow-up times and identify the drivers of the costs when comparing overall costs in these patients.

HER2 + patients incurred significantly higher charges vs. HER2- patients across all charge categories which was primarily driven by biologic therapy ($71,885 vs. $732; *p* < 0.0001). Patients who received neoadjuvant treatment after breast cancer diagnosis incurred the highest charges ($117,922), followed by those with metastatic disease ($101,969), and patients who received adjuvant treatment ($80,061).

Due to the variations in perspective, study population, year of cost basis, and duration of follow-up, for published breast cancer cost of illness studies, it is difficult to directly compare the costs for this study with existing literature. We did not observe previous studies that compared costs between BRCAm and BRCAwt or HER2 + and HER2- breast cancer patients; however, there are studies that have estimated costs by breast cancer stage and type of treatment. A recently published study by Blumen et al. estimated the overall per-patient allowed cost during the first year after breast cancer diagnosis to be $85,772 which is similar to the average mean charges observed in this study for BRCAm and BRCAwt ($85,982).[11] The Blumen study estimated costs by cancer stage and aggregated the costs across inpatient, outpatient, surgery, chemotherapy, radiation, prescription, and professional costs in a commercial claims database. The breast cancer treatment-related costs during the first year after diagnosis was $47,452 in the Blumen study vs. $41,277 in our study. In another study, Fu et al. estimated the per-patient medical costs during the 12 months after breast cancer diagnosis to be approximately $60,000 (2008 dollars) [12]. Other studies have estimated lifetime costs of breast cancer that have ranged from $36,926 (1984 values)[13] to over $100,000 (2003 values) [14].

With respect to treatment costs by type, Campbell and Ramsey, in a synthesis of published cost studies for breast cancer, noted that patients who received adjuvant therapy had significantly higher costs ($23,000–31,000) vs. those who did not.[4] Though not exactly comparable with our study due to differences in methodology (BRCA tested population), we observed significantly higher charges for neoadjuvant vs. adjuvant therapy ($117,922 vs. $80,061, *p* < 0.0001).

This study has a number of limitations. First, the results may not be generalizable since the results were for a single site (HCI) in Utah with a younger, Caucasian population. Some of the high treatment costs seen in this study may be a result of the younger age of the breast cancer cohort. Also, the community practice patterns may be different from what was observed at HCI, which is the only NCCN/NCI designated comprehensive cancer center in the Intermountain West region. Secondly, the study results were limited by the relatively small number of BRCAm patients (*n* = 134) vs. the BRCAwt (*n* = 682) group. Thirdly, the charges were aggregated across breast cancer disease stage, hence were not comparable to the more commonly published breast cancer cost studies that assessed costs by stage.

## Conclusions

Our assessment of the treatment charges for breast cancer at a single NCCN/NCI-designated comprehensive cancer center in the Intermountain West region demonstrates that BRCA mutation status was not associated with higher breast cancer charges, but were associated with significantly higher all-cause charges. However, HER2 + status was associated with higher breast cancer charges versus HER2- tumors and this was driven by the cost of biological agents. Breast cancer patients who initially received neoadjuvant treatment after diagnosis had significantly higher breast cancer related treatment charges than those who received adjuvant therapy. These economic differences by mutation, receptor and treatment setting are important to consider when evaluating the impact of targeted therapy on overall survival benefit.

## Data Availability

The datasets during and/or analyzed during the current study available from the corresponding author on reasonable request.

## Abbreviations

BRCA: Breast cancer
BRCAm: Breast cancer mutation
BRCAwt: Breast cancer wild-type phenotype
HER2+: Human epidermal growth factor receptor 2 positive
HER2-: Human epidermal growth factor receptor 2 negative
CMS: Center for Medicare & Medicaid Services
ER: Estrogen receptor
PR: Progesterone receptor
HCI: Huntsman Cancer Institute
NCCN: National Cancer Care Network
NCI: National Cancer Institute
